# Scattering angle resolved optical coherence tomography measures morphological changes in *Bacillus subtilis* colonies

**DOI:** 10.1117/1.JBO.27.12.126004

**Published:** 2022-12-30

**Authors:** Vikram Barauah, Shyon Parsa, Naail Chowdhury, Thomas Milner, Henry Grady Rylander

**Affiliations:** a The University of Texas at Austin, Biomedical Optics Lab, Department of Biomedical Imaging, Austin, Texas, United States; b UT Southwestern Medical School, Dallas, Texas, United States; c University of California Irvine, Beckman Laser Institute and Medical Clinic, Irvine, California, United States

**Keywords:** bacteria, optical coherence tomography, scattering angle resolved optical coherence tomography, scattering angle distribution

## Abstract

**Significance:**

An unmet need is recognized for early detection and diagnosis of neurological diseases. Many psychological markers emerge years after disease onset. Mitochondrial dysfunction and corresponding neurodegeneration occur before onset of large-scale cell and tissue pathology. Early detection of subcellular morphology changes could serve as a beacon for early detection of neurological diseases. This study is on bacterial colonies, *Bacillus subtilis*, which are similar in size to mitochondria.

**Aim:**

This study investigates whether morphological changes can be detected in *Bacillus subtilis* using scattering angle resolved optical coherence tomography (SAR-OCT).

**Approach:**

The SAR-OCT was applied to detect scattering angle distribution changes in *Bacillus subtilis.* The rod-to-coccus shape transition of the bacteria was imaged, and the backscattering angle was analyzed by recording the distribution of the ratio of low- to medium angle scattering (L/M ratio). *Bacillus* orientation at different locations in colonies was analytically modeled and compared with SAR-OCT results.

**Results:**

Significant differences in the distribution of backscattering angle were observed in *Bacillus subtilis* transitioning from rod-to-coccus shapes. In *Bacillus subtilis*, the C-parameter of the Burr distribution of the SAR-OCT-derived L/M ratio was significantly smaller in coccus compared with rod-shaped bacteria. SAR-OCT-derived L/M ratio varied with bacterial position in the colony and is consistent with predicted orientations from previous studies.

**Conclusions:**

Study results support the potential of utilizing SAR-OCT to detect bacterial morphological changes.

## 1 Introduction

Several studies have demonstrated that angular distribution of scattered light depends on cellular morphology and orientation. Polarized light is especially useful to investigate scattering changes that are correlated to cellular morphology and orientation, as reviewed by Tuchin.^1^ Dunn et al. showed that organelles on the scale of mitochondria can significantly contribute to cellular light scattering, with their volume fraction and morphology affecting the strength and angular distribution of backscattered light.^2^^,^^3^ Work reported by Su et al.^4^ suggests that mitochondria contribute to two-dimensional light scattering patterns of human B lymphoblastoid cells. Additionally, studies recording cellular light scattering with photomultiplier tubes have shown scattering is affected by morphologic changes in mitochondria associated with oxidative stress. Using optical scatter imaging, an increase was observed in the ratio of wide-to-narrow angle scattering that was concurrent with mitochondrial fragmentation, or a shift towards mitochondrial fission, during apoptosis.^5^^–^^7^ Furthermore, optical scattering changes corresponding to mitochondria were detectable within the first three hours of apoptosis, suggesting the potential of mitochondrial scattering properties as an early optical biomarker for tissue pathology. Thus, imaging approaches that can investigate morphology at an organelle scale, noninvasively, have potential to provide clinical value.

Standard optical coherence tomography (OCT) methodologies do not have the spatial resolution to directly image constituents on the scale of mitochondria, whereas variants of OCT coupled with robust image processing have been shown to be sensitive to subcellular phenomena. Speckle analysis of light scattered from cells has provided indications of increased intercellular motion, for example, that is concurrent with mitochondrial fission and apoptosis.^8^ Scattering angle resolved OCT (SAR-OCT) is a candidate approach to discriminate a shift to fission by detecting a change in the angular distribution of backscattered light. The SAR-OCT separates incident and backscattered light from the sample into discrete angular ranges by placing a pathlength multiplexing element (PME) in the sample path of the interferometer at a location conjugate to the pupil. For a PME with two discrete subapertures, four OCT subimages are generated of which two have degenerate path lengths. In this case, the ratio of intensities between the lowest and middle angular images (L/M ratio) is indicative of the ratio of smaller to larger backscattering angles.^9^^–^^12^

In this study, we used SAR-OCT to image *Bacillus subtilis* undergoing a rod-to-coccus transformation. When heated, *Bacillus* undergoes a change from its natural rod morphology with a size of 4 to 10  μm in length and 0.25 to 1.0  μm in diameter to a more circular coccus morphology with a length of 0.6 to 2.7  μm and a diameter of 0.4 to 1.8  μm.^13^^–^^15^ Because SAR-OCT is sensitive to light backscattering angle, we expect that the L/M ratio recorded from *Bacillus subtilis* will vary between the native rod to coccus morphology.

### 1.1 SAR-OCT

Although scattering angle resolved optical coherence tomography (SAR-OCT) instrumentation utilized in this study is described in detail by Gardner et al.,^15^ a brief overview is given here. The SAR-OCT instrumentation uses a narrow linewidth tunable laser source and is simple modification of standard swept-source OCT. As with conventional OCT, the sample path of the SAR-OCT interferometer uses an afocal scanning system with the pupil conjugate to lateral scanning mirrors. The SAR-OCT system utilized in this study uses a swept-source laser (1310 nm, ±70  nm; 100 kHz sweep rate) from Axsun, Inc. (Billerica, Massachusetts, United States). The system has a lateral resolution of 24.0  μm and an axial resolution of 11.7  μm in air.

SAR-OCT differs from conventional OCT through the positioning of a transparent PME into the sample path of the interferometer at a plane conjugate to the pupil of the afocal scanning system. The PME utilized in this study is radially angle-diverse and separates incident and backscattered light at lower and higher angles into four different pathlengths. The four pathlengths introduced by the PME correspond to hh, hl, lh, and ll, where h is high-angle and l is low-angle. Because hl and lh are degenerate or equal pathlengths, SAR-OCT instrumentation records the B-Scan sub-images that we label here as H (hh), M (hl and lh), and L (ll). Using a Fourier optics approach, Yin et al.^16^ derived analytical expressions for the SAR-OCT optical transfer functions for H (hh); M (hl and lh); and L (ll). The SAR-OCT transfer functions [Eq. (23) $H_{l,l}$, Eq. (24) $H_{h,h}$, and Eq. (25) $H_{h,l}$ and $H_{l,h}$ in Yin et al.] are the Fourier transforms of the point-spread function and give the complex amplitude ratio between spatial-oscillations in object and image planes. An important element in Yin et al.’s analysis is the sample backscattering angular diversity function [Eq. (6) in Yin et al.]. By assuming the backscattering angular diversity function has a Gaussian form, Yin et al. showed that the SAR-OCT L/M intensity ratio decreases with increased angular variance [σ in Yin et al., Eq. (27)] of backscattered light. Larger angular variance (σ) gives smaller SAR-OCT L/M ratios, whereas more narrow angular backscattering variance (i.e., small σ) give larger L/M intensity ratios.

## 2 Methods

### 2.1 *Bacillus subtilis* Colonies

**Fig. 1:**
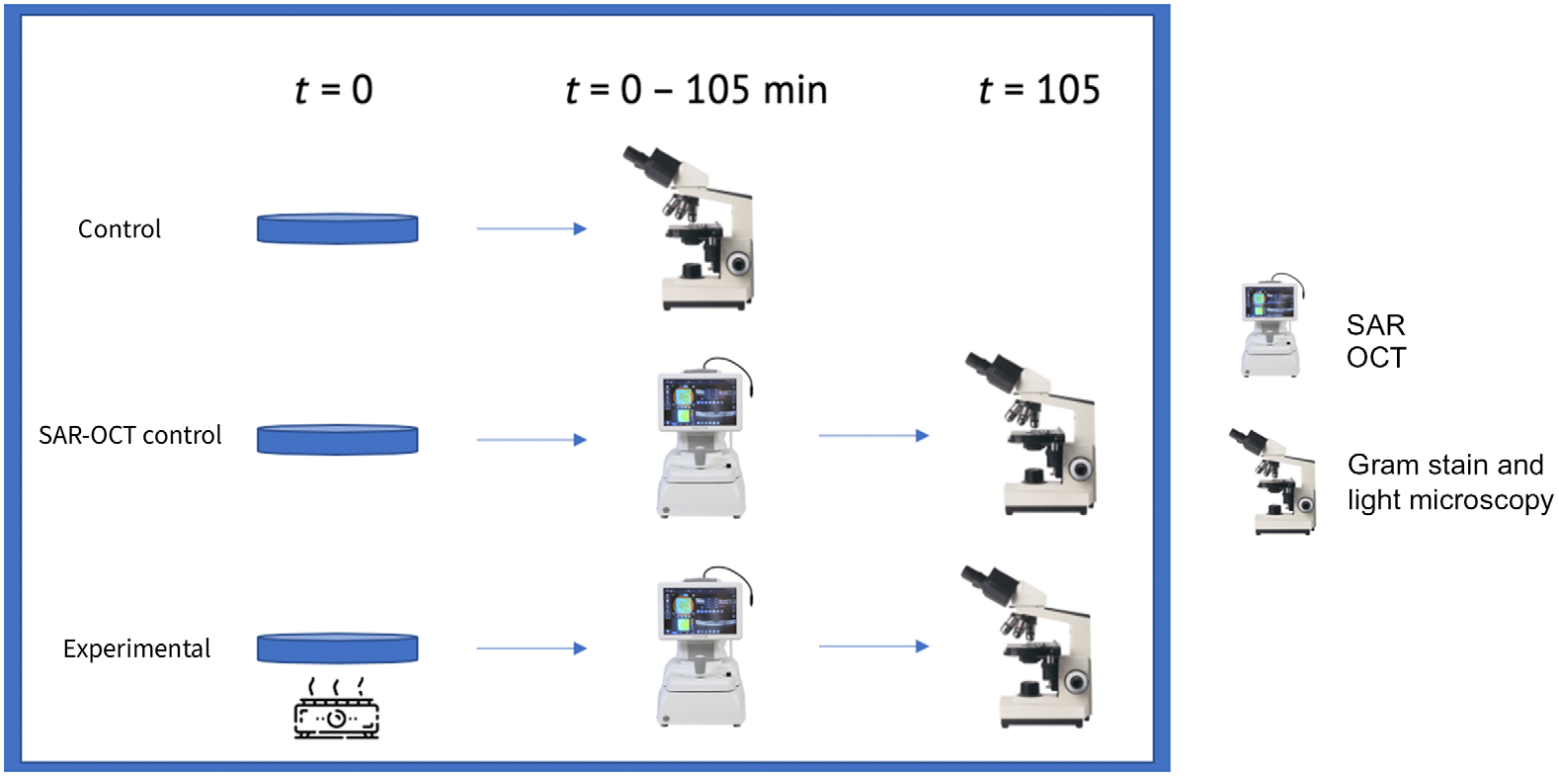
Bacterial imaging and processing. Each Petri dish represents three trials. Control plates were never exposed to SAR-OCT. The SAR-OCT control plates were never exposed to heat. Heating of the experimental plates were from t=0 throughout SAR-OCT imaging.

*Bacillus subtilis* was selected due to similar size to mitochondria (1 to 10 microns) and with analogous rod-to-coccus morphological transitions depending on temperature. Average thickness of bacterial colonies studied were 480 microns. *Bacillus* was cultured in five different Petri dishes to produce five distinct colonies. Prior to imaging, each dish was sampled/swabbed twice to create two sister colonies in two other dishes, which were used for gram staining and a control. Thus, a total of 15 Petri dishes were prepared (Fig. 1).

### 2.2 *Bacillus Subtilis* Imaging and Data Collection

Five imaging trials were conducted. In each trial, an experimental, SAR-OCT control, and gram stain control Petri dishes were imaged. Each of the dishes within a trial had *Bacillus* sister colonies. The first Petri dish was not subjected to temperature change and was gram stained and imaged using a light microscope to set a baseline for initial *Bacillus* morphology. The second dish was imaged by SAR-OCT while gradually being heated from room temperature (21°C) to 42°C over at least 100 min to induce a rod-to-coccus morphological change.^13^^,^^14^ The induction of rod-to-coccal morphology has been previously described and the time point selected allowed for sample-wide morphological changes without inducing cell wall thickening secondary to cross linking.^15^ Once SAR-OCT imaging was complete, the bacterial colonies were gram stained again and imaged via light microscopy to validate a transition from rod-to-coccus morphology occurred. The third Petri dish served as an imaging control and was subjected to the same temperature change as the second Petri dish and underwent gram staining but was not imaged via SAR-OCT. The third Petri dish was heated to 42°C while in contact with a sand bath. Sand was utilized to minimize movement of the Petri dish during the duration of the experiment and ensure consistent and repeatable SAR-OCT imaging throughout the experiment. Temperature of the bacterial colony surface was recorded using a noncontact infrared thermometer. Once the infrared thermometer indicated 42°C, a 105-min timer was initiated, as detailed in Burdett et al.’s imaging protocol.^15^

### 2.3 Analysis SAR-OCT Images of *Bacillus subtilis* Colonies

Image segmentation and fitting the L/M distributions was applied to SAR-OCT data to detect the rod-to-coccus transition. From SAR-OCT data, raw images were compiled into 512 (x) by 512 (y) by 160 (z) voxel B-scan stacks, which were large enough to capture individual colonies. Several colonies were captured in each Petri, providing at least 30 colony samples per trial. To separate the bacterial images from the Petri dish, Otsu’s thresholding was applied to SAR-OCT images of *Bacillus* colonies. After sampling only pixels relating to the *Bacillus*, images were flattened and the L/M ratio was calculated over every pixel in the *Bacillus* samples. Bacterial colonies were analyzed over five trials, incorporating hundreds of cells that, while directly analyzed in gram stain, are analyzed as whole colonies during SAR-OCT intensity analysis.

After segmentation, L/M intensity ratios for cocci were compared to values before the rod-to-coccus transition. To further differentiate between rod and coccus morphologies, a histogram of L/M ratios was compiled by considering active pixels within a *Bacillus* colony.

### 2.4 Analysis of Gram Stain Images of *Bacillus subtilis* Colonies

Changes in average bacterial length of a colony in images of the gram stains were used to validate a rod-to-coccus transition occurred. Bacterial length was manually extrapolated based on overall image scale. Length was calculated for all bacteria within a randomly selected quadrant of the gram stain image (Fig. 3).

### 2.5 Analysis of *Bacillus* Orientation

The three-dimensional architecture of colonies of rod-shaped bacterial (Vibrio Cholera, E. Coli, *Bacillus subtilis*) has been studied from both theoretical and microscopic perspectives. Bacteria placed on agar form a monolayer biofilm initially with the long axis of the bacteria parallel to the surface of the agar plate. As the biofilm grows, the bacteria begin to stack in the center with their long axis perpendicular to the agar plate. Eventually the colony assumes a three dimensional hill-like structure with bacteria at the surface oriented parallel to the agar plate and bacteria in the depth of the center oriented perpendicular to the agar plate. Studies have demonstrated, using confocal imaging and computational modeling, that bacterial orientation in a colony varies from surface to bottom and from center to periphery. Thus, distribution of L/M ratios are expected to vary with location in a colony. Literature shows that *Bacillus* at the center and toward the bottom and middle of the colony by depth will be oriented with the long axis of the bacteria largely perpendicular to the plane of the agar plate.^17^^–^^22^ Alternatively, *Bacillus* on the colony surface and towards the peripheral edges will be oriented with their long axis parallel to the surface agar in the plate.

Based on basic scattering theory and Yin et al.’s results, these documented variations in cell orientation are expected to impact SAR-OCT L/M ratios at different regions in the *Bacillus* colonies. To investigate whether SAR-OCT can detect changes in bacterial orientation documented by Warren et al.,^17^ Panning windows (15×15) were applied to SAR-OCT B-scan vertical slices of the colonies. L/M ratios were sampled from and averaged within the panning window. Panning windows were applied to every pixel in the colony images.

## 3 Results

### 3.1 *Bacillus subtilis* Whole Colony Gram Stain Analysis

**Fig. 2:**
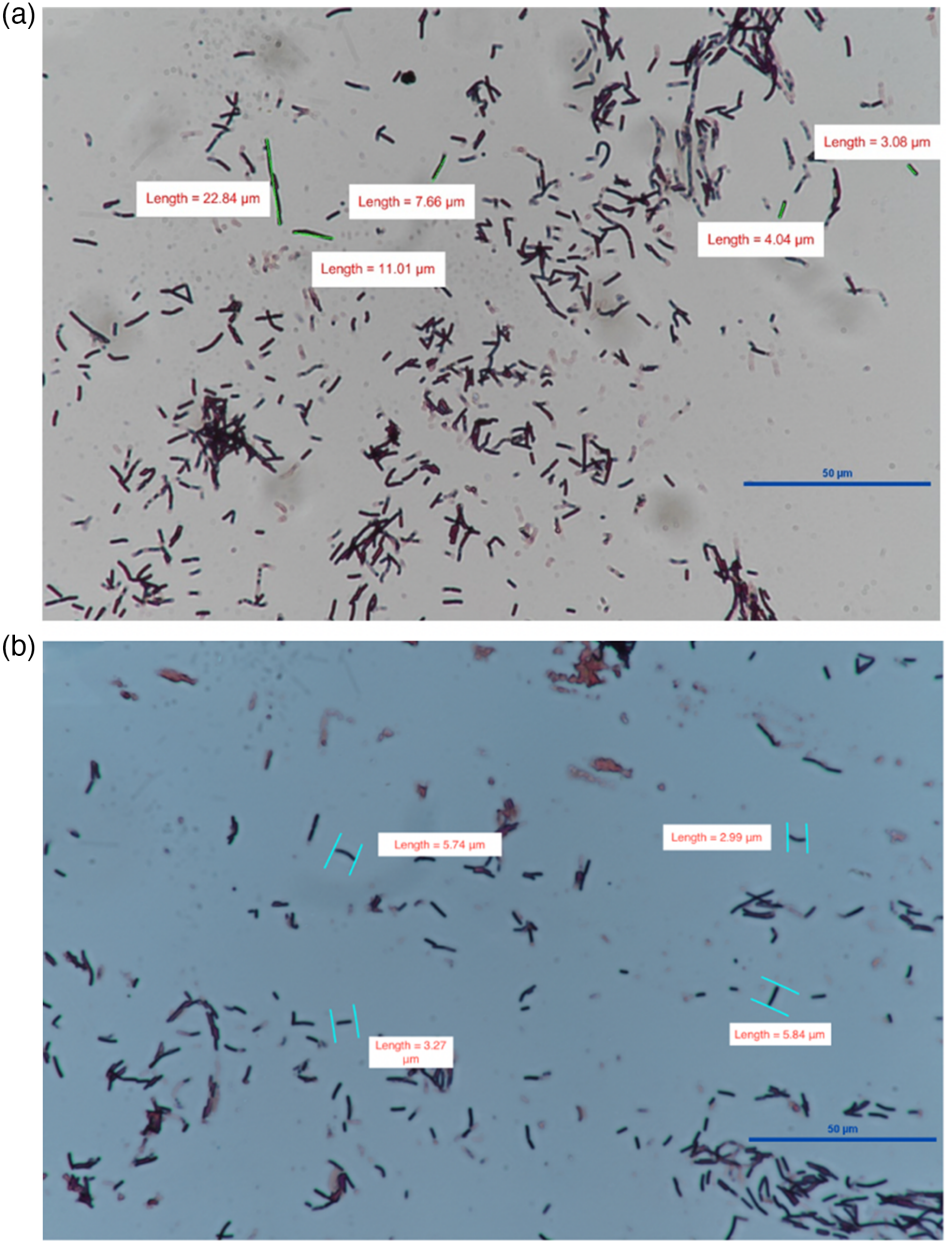
Bacterial gram stains: (a) image of a gram stain of colonies with no heating and (b) image of a gram stain of colonies after heating. Average bacterial length before and after heating were 7.42±0.6  microns and 4.8±0.8  microns, respectively (p=0.038). The scale bar in both images is 50  μm.

Microscopy of bacterial colonies confirmed consistent controls and initial conditions across all five trials. Bacterial colonies demonstrate a rod-shaped morphology in dish one in all five trials and no significant difference in average bacterial length or bacterial clustering density between dishes two and three over all five trials. Gram stains conducted on bacterial colonies confirm the predicted rod-to-coccus transition in bacterial morphology after heating. On average, bacterial length before heating was 7.42±0.6  microns and 4.8±0.8  microns after heating (p=0.038), and bacterial orientation was random on the gram stains (Fig. 2).

### 3.2 *Bacillus subtilis* Whole Colony L/M Ratios

A significant change in L/M intensity ratio was observed preheating, 1.48 (mean) ± 0.608 (standard deviation) versus post heating, 1.2086±0.6679 (s) with a T-test p=0.032. When a composite L/M ratio image was formed and its intensity histogram was fit to the Burr distribution, the C-parameter (representing approximate width of the distribution could be applied to discern between rod and coccus *Bacillus*: average C-parameter for preheating is 4.1±0.33 versus postheating is 5.4±0.27 (p=0.047). While individual L/M ratios alone could not significantly distinguish between rod and coccus bacterial colonies, C-parameters derived from Burr distributions provided statistically significant and robust discrimination.

### 3.3 *Bacillus subtilis* Orientation Analysis

**Fig. 3:**
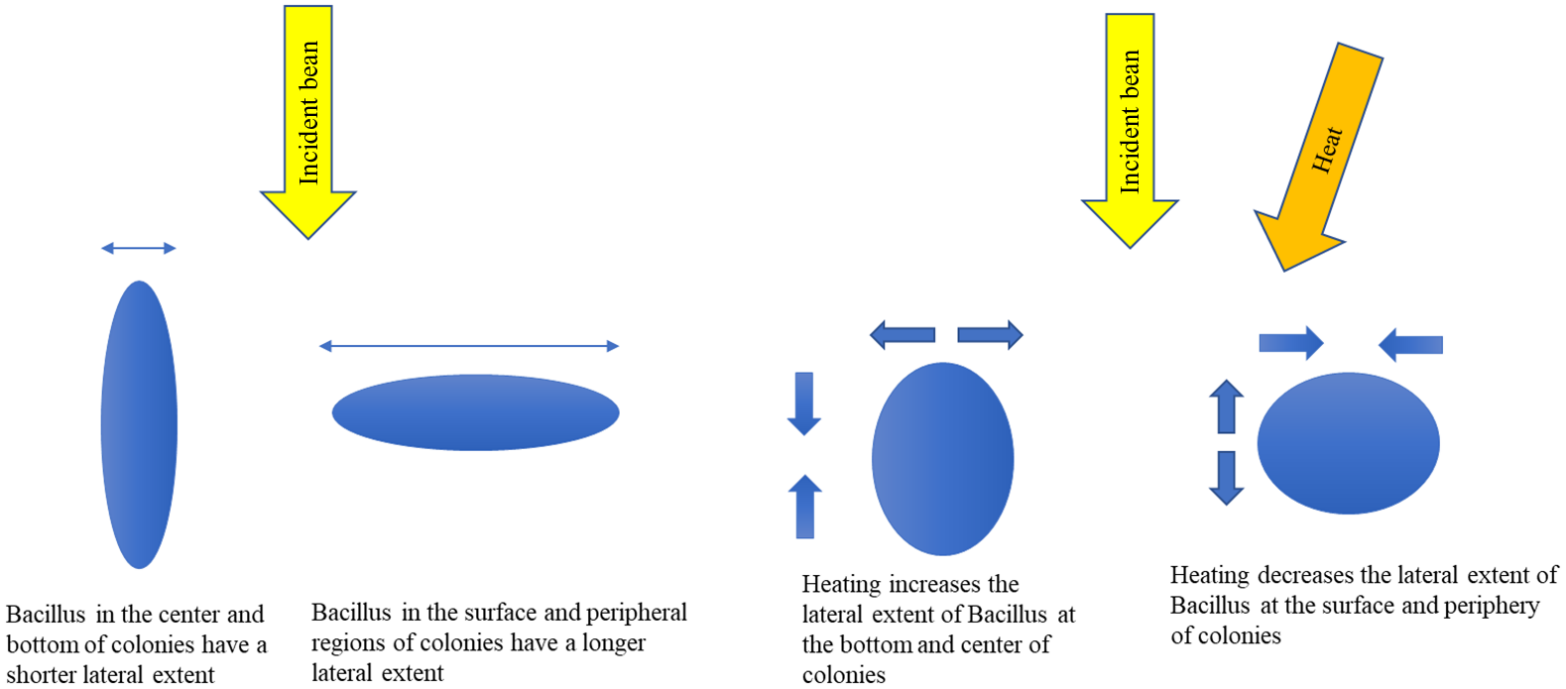
SAR-OCT L/M intensity ratio and relationship with *Bacillus* orientation.

Four factors are recognized where differences in *Bacillus* orientation may affect the SAR-OCT L/M ratio. The conditions are illustrated in Fig. 3.

1. Vertical location in colony: bacteria at surface (perpendicular to beam) versus bacteria at the bottom of colony (upright/parallel to beam). Here, the L/M ratio of the bacteria at the surface of the colony is expected to be larger than L/M of bacteria at the center and bottom of the colony.
2. Lateral location in colony: bacteria at the center of colony (upright/parallel to beam) versus bacteria at periphery (perpendicular to beam). The L/M ratio of bacteria in the center of the colony is less than L/M of bacteria in the periphery.
3. Heating and vertical location in colony: after heating, bacteria at surface shrink in lateral extent perpendicular to beam versus bacteria at bottom of colony increase in radius and thus lateral extent perpendicular to the beam. Thus, the L/M ratio of bacteria at the surface of the colony decreases after heating, whereas the L/M of bacteria at the bottom of the colony are expected to increase after heating. After heating, *Bacillus* shrink along the long axis and widen along the short axis. Thus, the lateral extent of *Bacillus* originally perpendicular to the image beam, such as *Bacillus* on the colony surface, are expected to shrink. In contrast, the lateral extent of *Bacillus* originally parallel to the image beam, such as *Bacillus* on the bottom of the colony, will increase. Because the L/M ratio increases with the lateral extent of the scatterer, the L/M of *Bacillus* on the surface of the colony is predicted to decrease and the L/M ratio of *Bacillus* in the bottom of the colony is predicted to increase.
4. Heating and lateral location in colony: bacteria at the center of the colony increase in radius and thus lateral extent perpendicular to the beam vs. bacteria at the periphery shrink in lateral extent perpendicular to beam after heating. The L/M ratio of the *Bacillus* at the center of the colony is expected to increase while the L/M ratio of *Bacillus* at the periphery is predicted to decrease after heating according to basic scattering theory and Yin et. al.’s result.

Based on basic scattering theory and Yin et al.’s results, SAR-OCT L/M intensity increases with the lateral spatial extent of the scatterer with respect to the incident beam. Thus, *Bacillus* in different locations in the colony and with different morphologies after heating will have different L/M distributions.

1. Bacteria at surface (perpendicular to beam) have a higher L/M versus bacteria at bottom of colony (upright/ parallel to beam).
2. Bacteria at periphery of colony (upright/ parallel to beam)) have a higher L/M versus bacteria at the center (perpendicular to beam).
3. After heating, bacteria at the bottom and center of the colony will experience an increase in L/M and bacteria at the surface and periphery will have a decrease in L/M.

**Fig. 4:**
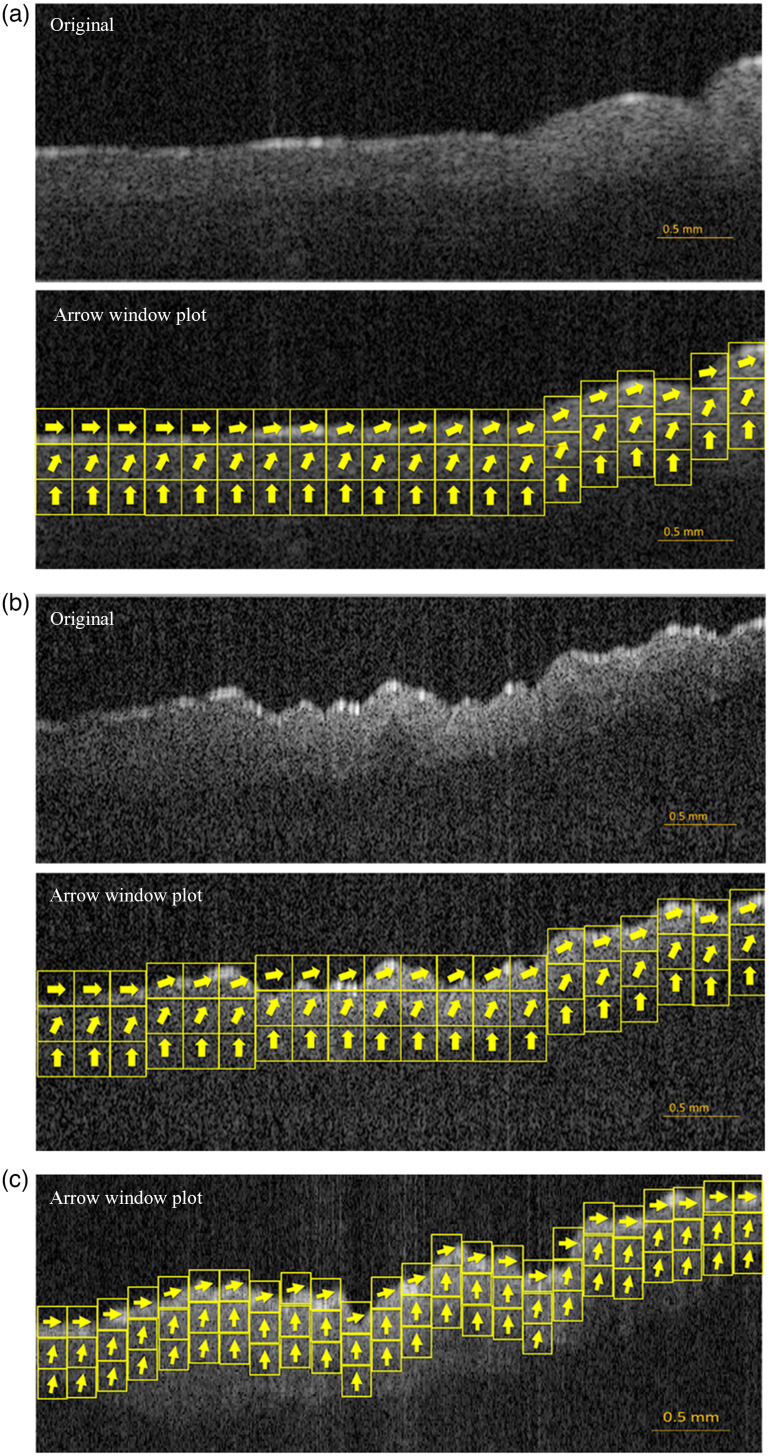
SAR-OCT B-scan-derived images of *Bacillus* orientation in a colony. L/M ratio varies from 1.03 to 1.44 across all bacterial colonies. Assuming 1.03 (L/M) corresponds to a 90 deg orientation and 1.44 to be 0 deg from the agar plate surface, respectively, the arrow window plots were overlaid on the bacterial colony to indicate predicted *Bacillus* orientation. An empirically derived equation gives *Bacillus* orientation (Θ) under these imaging conditions: (L/M−1.03)/0.41 = cos(Θ). Panels (a)–(c) are B-scans from different *Bacillus* colonies, with (a) and (b) having the raw B-scan coupled with L/M arrow window plots. L/M ratios are highest at the surface of the colonies.

Panning window sampling and averaging of the L/M ratio across *Bacillus* colony B-scans demonstrated that as expected the L/M ratio varied based on location within the colony—surface versus bottom and center versus periphery. L/M varied from 1.44, at the colony surface, to 1.03 at the bottom and center of the colony. Considering that long axis of bacteria at the colony surface are predicted to be perpendicular to the direction of incoming OCT light and the bacteria in the bottom are predicted to be parallel to the image beam, L/M values were translated to angles based on an empirically derived equation: (L/M-1.03)/0.41 = cos(Θ). Here, the offset value 1.03 represents the minimum L/M value in the experiment and 0.41 is the observed variation of L/M values and is used to scale angles ranging from 0 deg to 90 deg relative to the plate surface. Using this empirical equation, several images (Fig. 4) were computed indicating *Bacillus* orientation.

### 3.4 Variance in L/M Ratios after Heating by Colony Location

Results of L/M change following heating of *Bacillus* colonies also followed qualitative predictions from basic scattering theory and Yin et al.’s results and are summarized in Table 1.

**Table 1:** L/M ratios postheating. Results follow predicted increases and decreases in L/M ratio for *Bacillus* at the bottom and center regions of a colony and *Bacillus* at the surface and periphery of the colony, respectively. All changes were statistically significant p<0.05.

	Surface	Bottom	Center	Periphery
Expected postheat versus preheat L/M change	Decrease	Increase	Increase	Decrease
Average postheat versus preheat L/M change	−0.152	0.148	0.153	−0.142
Average percent postheat versus preheat L/M change	−10.85%	+14.1%	+14.57%	−10.14%

## 4 Discussion

From the *Bacillus* experiment, the hypothesis that SAR-OCT is sensitive to cellular morphological changes (and orientation) was confirmed. Predicted trends derived from the literature (Warren et al.) and simple scattering theory and Yin et al.’s analysis were confirmed that a change in scattering angle accompanies a change in morphology.^17^

Experiments with *Bacillus* aimed to demonstrate that SAR-OCT can detect changes in scattering from scattering centers at the subcellular scale (e.g., mitochondria). Whereas several intracellular morphological changes are recognized that can contribute to change in light back scattering angle distribution from tissues, detecting changes stemming from mitochondria may provide clinical utility due to the several neurodegenerative diseases associated with mitochondrial dysfunction. A significant challenge, however, with connecting the specific *Bacillus* morphological change with backscattering angle variation stems from the specifics of bacterial stacking and organization within colonies. Simple scattering theory and Yin et al.’s results suggest that the SAR-OCT L/M intensity ratio will decrease (increase) as lateral spatial extent of the scattering center becomes smaller (larger). However, without knowing (or predicting) bacterial orientation within a colony, it is difficult to associate an increase or decrease in scattering angle range with a shift from rod to coccus shape. Thus, including a model of bacterial orientation within *Bacillus* colonies was necessary to interpret the observed morphological changes.

We used results presented by Warren et al., which modeled forces of surface tension and bacterial adhesion in colonies, to generalize bacterial orientation in *Bacillus* colonies. Warren et al.^17^ further validated their model with microscope images of individual bacterial orientations at different locations in colonies. Based on this work, *Bacillus* at the center of the colony are predicted to be more upright and with their long axis parallel to the incoming OCT beam than those at the periphery. Additionally, *Bacillus* at the surface layers of a colony are oriented flat with their long axis perpendicular to the incoming OCT beam versus bacteria at the bottom that are oriented parallel to the beam. These differences in cellular orientation lead to different predicted values in the L/M ratio and changes in the L/M ratio after a heat-induced rod-to-coccus transformation at different spatial regions in a colony. Resulting average L/M ratios calculated across colonies before and after heating are consistent with predicted trend changes (Table 1). Percentage changes were also statistically significant and >10% of the original L/M values, indicating good classification power. Further work can be conducted to generate predicted theoretical L/M values with detailed simulations of *Bacillus* optical properties, but the general trend of L/M changes predicted for each region in a colony matched the corresponding experimental L/M values. Thus, this study demonstrates that SAR OCT is sensitive to cellular morphology under eight experimental conditions: colony surface, colony bottom, colony center, colony periphery, and rod-to-coccus changes in the four regions studied. The ability of SAR-OCT to provide information related to the orientation of the rod-shaped bacteria may also have significance in studying other tissues.

To further validate the experimental results, other bacterial cell types, such as gram negative and gram positive bacteria, can be sampled and imaged in future work to corroborate SAR-OCT sensitivity to detect morphological change. A challenge with studying cell types other than fibroblasts, such as neurons for example, is generating homogenous constructs with sufficient depth to be imaged by OCT. In work reported here, this depth was generally four pixels for sufficient signal-to-noise ratio.

Results of this study suggest possible avenues of further research. The instrumentation and analysis can be extended by considering different PME designs that enable either a wider range or a more highly resolved range of back scattering angles that might improve the discerning power of the approach. Furthermore, finite element or discrete dipole scattering model calculations could be applied to the SAR-OCT sample path and interface with Yin et al.’s Fourier optics model to generate predicted L/M ratio values at different regions of *Bacillus* colonies to provide another benchmark for experimental results presented here.

## 5 Conclusion

In this study, we demonstrate a novel method using SAR-OCT for studying orientation and shape change in *Bacillus subtilis* colonies. By analyzing the ratio of intensities of low-angle to medium-angle backscattered OCT light, *Bacillus subtilis* of different shapes were distinguished with statistical significance. The detection of a bacterial rod-to-coccus shape change by analysis of backscattered light can provide approaches for noninvasive early detection of tissue conditions associated with cellular shape or state changes.
